# Metagenomic Next-Generation Sequencing for Pulmonary Tuberculosis Diagnosis and Infection Risk Factor Analysis in AECOPD Patients: A Single-Center Retrospective Study

**DOI:** 10.3390/jcm15124507

**Published:** 2026-06-10

**Authors:** Chao He, Hua Zou, Ziyang Jiang, Yi Zhou, Binwu Ying

**Affiliations:** 1 Department of Laboratory Medicine, Clinical Laboratory Medicine Research Center, Sichuan Clinical Research Center for Laboratory Medicine, West China Hospital of Sichuan University, Chengdu 610041, China; hechao@wchscu.cn (C.H.); zyjiang47@wchscu.cn (Z.J.); zhouyi2011@wchscu.cn (Y.Z.); 2 Department of Laboratory Medicine, Chongqing Health Center for Women and Children, Women and Children’s Hospital of Chongqing Medical University, Chongqing 400016, China; 115225@hospital.cqmu.edu.cn

**Keywords:** acute exacerbation of chronic obstructive pulmonary disease, metagenomic next-generation sequencing, tuberculosis, diagnostic performance, risk factors

## Abstract

**Background**: Pulmonary tuberculosis (TB) is a significant trigger of acute exacerbations of chronic obstructive pulmonary disease (AECOPD), so its timely and accurate diagnosis is essential. Also, the risk factors for TB occurrence in this population remain unclear. This study aimed to evaluate the performance of metagenomic next-generation sequencing (mNGS) for TB diagnosis in AECOPD patients, as well as to identify the associated risk factors. **Methods**: A retrospective observational cohort of 659 AECOPD patients with suspected pulmonary infection was enrolled. The microbial cell-free nucleic acids in bronchoalveolar lavage fluid samples were extracted and subjected to mNGS detection. The clinical data for each patient were collected from the hospital information system. The statistical analyses were performed with SPSS version 25.0. **Results**: A total of 170 cases, included for final analyses, were categorized into TB (*n* = 41), bacterial infection (*n* = 73), and non-infective control (*n* = 56) groups. Among these groups, the TB group had the highest intensive care unit (ICU) admission rate (46.34%) and longest median hospital stay (19.50 days) (*p* < 0.01). For TB diagnosis, mNGS demonstrated a greater sensitivity (86.00%), a lower specificity (93.30%), and a higher area under the curve (AUC, 0.877) than TB-DNA detection (70.21%, 100%, 0.848, respectively) and Xpert *Mycobacterium tuberculosis*/rifampicin (MTB/RIF) assay (63.83%, 100.00%, 0.870, respectively). Notably, mNGS identified the bacterial or viral co-infections in 18.00% of TB cases. Furthermore, the stringently mapped read number determined by mNGS showed a positive correlation with ICU admission rate (r = 0.76) and in-hospital mortality (r = 0.77). The lower body mass index (BMI) and reduced natural killer (NK) cell count were identified as the independent risk factors in the TB group (both *p* < 0.05). **Conclusions**: For the diagnosis of pulmonary TB in AECOPD patients, mNGS demonstrated comparable performance to TB-DNA detection and Xpert MTB/RIF assay, and also mNGS identified co-infections. In addition, a lower BMI and reduced NK cell count were identified as the independent risk factors for TB occurrence in this cohort.

## 1. Introduction

Chronic obstructive pulmonary disease (COPD) is the third leading cause of death worldwide, affecting millions of individuals, and its prevalence is projected to rise to 592 million cases by 2050 [1]. Acute exacerbations of COPD (AECOPD) can accelerate lung function decline and impair health-related quality of life [2]. AECOPD events are the primary driver of COPD’s overall clinical and socioeconomic burden, as they substantially increase global healthcare expenditure, hospital readmission rates, and long-term care costs. Among the patients hospitalized for severe AECOPD, in-hospital mortality was reported to be approximately 11.46% [3]. In high-risk populations, the risk of death is approximately three times higher within 90 days after hospital discharge, and the cumulative mortality rate at two years may be as high as 50% [4,5].

AECOPD is most commonly triggered by respiratory tract infections [6], which involve diverse pathogens, such as bacteria [7], viruses [8], and Mycoplasma species [9]. Among these pathogens, *Mycobacterium tuberculosis* remains a global public health challenge with a substantial disease burden. According to the 2025 World Health Organization (WHO) Global Tuberculosis Report, an estimated 10.7 million people fell ill with TB, and approximately 1.23 million deaths were attributed to TB worldwide in 2024 [10]. The significant clinical concern arises from the robust association between COPD and a heightened risk of tuberculosis (TB) (odds ratio [OR] = 1.0008, 95% confidence interval [CI] = 1.0001–1.0014, *p* = 0.015) [11]. A systematic review of 711,389 participants reported that COPD had a 1.44~3.14-fold higher incidence of TB compared with those without COPD [12]. Notably, the patient’s mortality doubled in cases where COPD exacerbation was induced by TB (OR = 2.2, 95% CI = 1.2–4.1) [13]. Therefore, the accurate diagnosis of TB is crucial for improving the prognosis of AECOPD patients.

Currently, the diagnostic approaches for TB, e.g., acid-fast staining (AFS), mycobacterial culture, polymerase chain reaction (PCR), Xpert *M. tuberculosis*/rifampicin (MTB/RIF) assay, and interferon-gamma release assays (IGRA), are widely used in clinical laboratories [14]. However, these methods have notable limitations in clinical practice. AFS has a very low sensitivity. Mycobacterial culture is time-consuming and not suited for rapid etiological diagnosis [15]. Targeted pathogen testing, such as TB-DNA detection and Xpert MTB/RIF assay, cannot identify the unexpected or co-existing microorganisms [16]. IGRA cannot distinguish latent from active tuberculosis infection [17]. In recent decades, metagenomic next-generation sequencing (mNGS), a non-targeted and broad-spectrum pathogen detection technology, has emerged as a promising approach for infection diagnosis [18]. Compared with targeted nucleic acid-based assays, mNGS enabled the simultaneous detection of a variety of pathogens. Recent findings demonstrated the good clinical utility of mNGS in pulmonary infection, particularly for identifying co-infection and difficult-to-culture pathogens [19,20], whereas there is limited research regarding its efficacy in AECOPD patients.

On the other hand, several factors, such as immune dysregulation [12], airway obstruction [21,22], and glucocorticoid usage [23], have been reported to contribute to TB occurrence in COPD patients. Some studies concentrated on the usage of pharmacological agents, especially inhaled corticosteroids and β-receptor agonists [24,25]. Additional evidence indicated that the baseline characteristics (e.g., age, male, diabetes) were independent risk factors for TB in COPD patients [26]. Nevertheless, important gaps remain in the risk factor analyses of TB among the patients with advanced chronic airway disease, including the limited number of available studies, insufficient disease phenotype stratification, and inadequate population- and region-specific evidence [12,25]. Therefore, identifying the specific risk factors within the local patient population is paramount.

The present study aimed to compare the diagnostic performance of mNGS with that of conventional diagnostic methods for pulmonary TB, and also to identify the associated risk factors in a retrospective cohort of AECOPD patients.

## 2. Materials and Methods

### 2.1. Patient Enrollment and Data Collection

**Figure 1:**
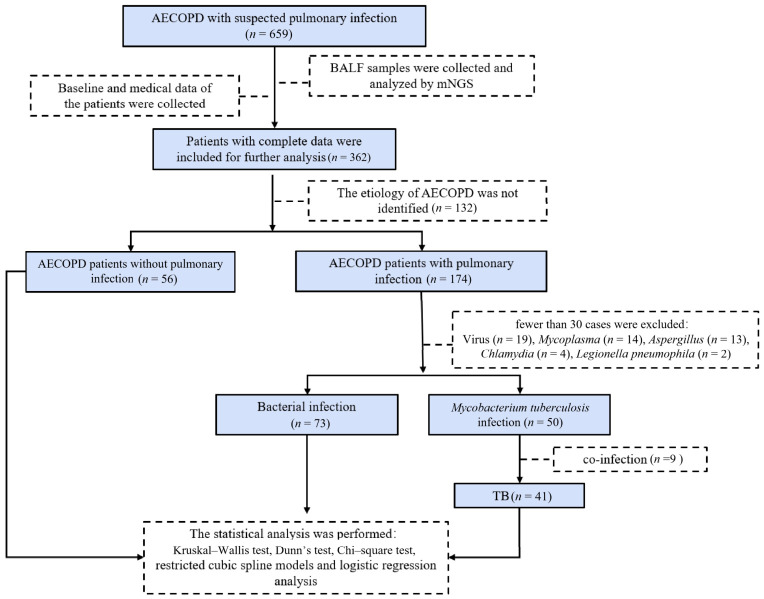
Flowchart of study design and case enrollment. Abbreviations: AECOPD—acute exacerbations of chronic obstructive pulmonary disease; TB—tuberculosis; BALF—bronchoalveolar lavage fluid; mNGS—metagenomic next-generation sequencing.

The diagnosis of AECOPD and pulmonary infection was established with reference to the 2023 Global Initiative for Chronic Obstructive Lung Disease (GOLD) guidelines [27] and the Official American Thoracic Society Clinical Practice Guidelines [28]. The study flowchart is presented in Figure 1. Initially, 659 AECOPD patients with suspected pulmonary infection admitted between December 2022 and November 2025 were enrolled. After excluding the cases with incomplete clinical data, undefined etiology, or those belonging to the subgroups with fewer than 30 cases, the remaining cases were categorized into three groups for final analyses: a TB group (*n* = 41), a bacterial infection group (*n* = 73), and a control group (*n* = 56). The classification of the TB, bacterial, and non-infective control groups was based on the comprehensive evaluation of clinical manifestation, epidemiology, imaging findings, and laboratory indicators.

### 2.2. Sample Collection and mNGS Assay

Bronchoalveolar lavage fluid (BALF) samples from AECOPD patients with suspected pulmonary infections were collected according to European Respiratory Society guidelines [29]. From 800 μL BALF aliquots, nucleic acids were extracted using the TIANamp Micro DNA Kit (Tiangen Biotech Co., Ltd., Beijing, China) and the QIAamp Viral RNA Mini Kit (QIAGEN GmbH, Hilden, Germany), respectively. RNA was reverse-transcribed into cDNA using SuperScript II Reverse Transcriptase (Thermo Fisher Scientific, Waltham, MA, USA), and double-stranded DNA was synthesized with DNA Polymerase I (Enzymatics Inc., Beverly, MA, USA). Library preparation (fragmentation, end repair, adapter ligation, PCR amplification) and sequencing were performed by Beijing Genomics Institute Genomics (Shenzhen, China) on the MGISEQ-2000 platform (MGI Tech Co., Ltd., Shenzhen, China). The bioinformatics pipeline included quality control and low-quality read filtering via Trimmomatic v0.39 (Usadel Lab, RWTH Aachen University, Aachen, Germany). The high-quality reads were mapped to microbial databases for pathogen identification. [18].

All samples underwent a standardized quality control pipeline, and only those meeting predefined sequencing quality criteria were included in the downstream analysis. For pathogen identification, a sample was considered positive when pathogen-specific reads were detected after quality filtering and the removal of human-derived sequences, and the aligned reads showed high-confidence matches to reference microbial genomes in the curated database. For example, a sample was defined as “TB-positive” when ≥ 1 unique reads of MTB were detected.

### 2.3. Statistical Analysis

Statistical analyses were performed using IBM SPSS Statistics (version 25.0; IBM Corp., Armonk, NY, USA). Categorical variables were described as frequency (percentage). Continuous variables were tested for normality (using the Shapiro–Wilk test). Non-normally distributed variables were expressed as medians (interquartile range, IQR). Categorical variables were analyzed using the chi-square test, as appropriate. The Kruskal–Wallis test was used for comparisons among three groups, and Dunn’s test with Bonferroni correction was performed for pairwise comparisons. Restricted cubic spline and logistic regression analyses were performed using R software (version 4.3.3; R Foundation for Statistical Computing, Vienna, Austria). All tests were two-sided, and a *p*-value < 0.05 was considered statistically significant.

## 3. Results

### 3.1. Demographic and Clinical Characteristics of the Cases Included

As summarized in Table 1, the TB group displayed a distinct clinical profile characterized by a significantly lowest body mass index (BMI) (19.96 [17.19–22.49] kg/m^2^), longest hospital stay (19.50 [14.25, 24.00] days), highest rate of ICU admission (46.34%), and longest ICU stay (0.00 [0.00, 10.75] days) (all *p* < 0.01). No significant inter-group differences were found in in-hospital mortality, medication history, or comorbidity (Table 1, Supplemental Figure S1, all *p* > 0.05).

**Figure 2:**
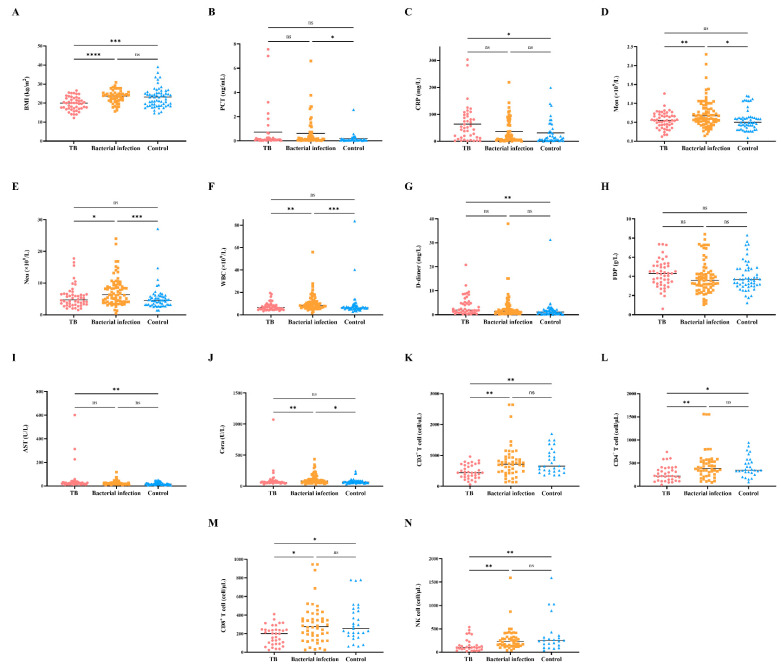
Differences in clinical and laboratory indicators among TB, bacterial infection, and control groups. (**A**) Comparison of BMI among the three groups. (**B**) Comparison of PCT levels among the three groups. (**C**) Comparison of CRP levels among the three groups. (**D**) Comparison of Mon count among the three groups. (**E**) Comparison of Neu count among the three groups. (**F**) Comparison of WBC count among the three groups. (**G**) Comparison of D-dimer levels among the three groups. (**H**) Comparison of FDP levels among the three groups. (**I**) Comparison of AST levels among the three groups. (**J**) Comparison of Crea levels among the three groups. (**K**) Comparison of CD3^+^ T Cell count among the three groups. (**L**) Comparison of CD4^+^ T Cell count among the three groups. (**M**) Comparison of CD8^+^ T Cell count among the three groups. (**N**) Comparison of NK Cell count among the three groups. * *p <* 0.05, ** *p <* 0.01, *** *p* < 0.001, **** *p <* 0.0001. ns, not significant. Abbreviations: TB—tuberculosis; BMI—body mass index; PCR—procalcitonin; CRP—C-reactive protein; Mon—monocyte; Neu—neutrophil; WBC—white blood cell; FDP—fibrin(ogen) degradation products; AST—aspartate aminotransferase; Crea—creatinine; T Cell—T lymphocyte; NK cell—natural killer cell.

Significant differences were observed across multiple laboratory parameters. The levels of procalcitonin (PCT, *p* = 0.02), C-reactive protein (CRP), and D-dimer (both *p* = 0.01) differed among the three groups, as did the counts of monocytes, neutrophils, white blood cells (WBC), T/natural killer (NK) cell subsets, and the levels of fibrinogen, fibrin(ogen) degradation products (FDP), aspartate aminotransferase (AST), and creatinine (Crea) (Figure 2B–J, all *p* < 0.01). Other parameters showed no significant differences (Supplemental Table S1). Post hoc analyses confirmed that the TB group had a significantly lower BMI (Figure 2A) and markedly reduced counts of CD3^+^/CD4^+^/CD8^+^ T and NK cells (Figure 2K–N) relative to the other two groups (all *p* < 0.01).

Radiologically, the TB group exhibited the highest frequencies of mediastinal involvement (66.00%) and cavitary changes (23.42%) (Supplemental Figure S2A,B). Pathological necrosis was a key feature of the TB group (71.40%, Supplemental Figure S2C) and significantly correlated with TB diagnosis (r = 0.64, Supplemental Figure S2D).

### 3.2. Overall Performance of mNGS in TB Diagnosis

**Figure 3:**
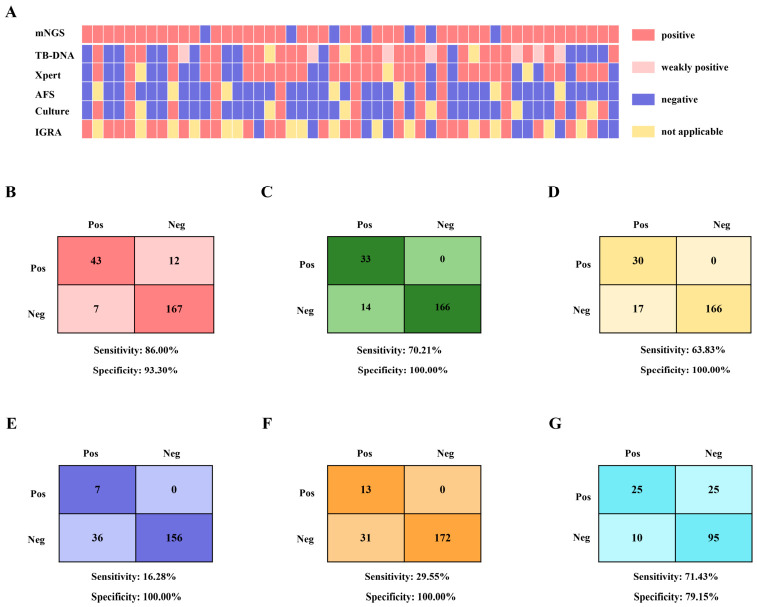
Diagnostic efficacy of different methods for TB in AECOPD patients. (**A**) Results of mNGS, TB-DNA detection, Xpert MTB/RIF, AFS, culture, and IGRA in 50 TB patients. (**B**) The diagnostic efficacy of mNGS. The numbers in the matrix represent the counts of true-positive (*n* = 43), false-positive (*n* = 12), false-negative (*n* = 7), and true-negative (*n* = 167). (**C**) The diagnostic efficacy of TB-DNA detection. The numbers in the matrix represent the counts of true-positive (*n* = 33), false-positive (*n* = 0), false-negative (*n* = 14), and true-negative (*n* = 166). (**D**) The diagnostic efficacy of Xpert MTB/RIF. The matrix showed 30 true-positive, 0 false-positive, 17 false-negative, and 166 true-negative cases. (**E**) The diagnostic efficacy of AFS. The counts displayed in the matrix represent true-positive (*n* = 7), false-positive (*n* = 0), false-negative (*n* = 36), and true-negative (*n* = 156) classifications. (**F**) The diagnostic efficacy of the culture. The matrix comprised 13 true-positive, 0 false-positive, 31 false-negative, and 172 true-negative results. (**G**) The diagnostic efficacy of IGRA. The numbers represent the numbers of cases classified as true positive (*n* = 25), false positive (*n* = 25), false negative (*n* = 10), or true negative (*n* = 95). Abbreviations: TB—tuberculosis; AECOPD—acute exacerbations of chronic obstructive pulmonary disease; mNGS—metagenomic next-generation sequencing; AFS—acid-fast staining; IGRA—interferon-gamma release assays; Pos—positive; Neg—negative.

**Figure 4:**
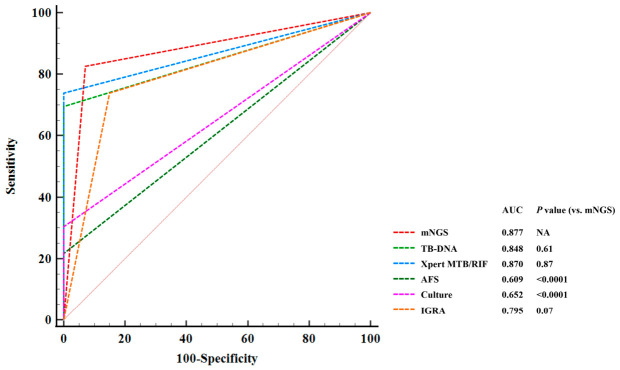
ROC curve analysis of multiple detection modalities in tuberculosis diagnosis. The pale red line represents the performance of a random classifier (AUC = 0.50). Abbreviations: ROC—receiver operating characteristic; mNGS—metagenomic next-generation sequencing; TB—tuberculosis; AFS—acid-fast staining; IGRA—interferon-gamma release assays; MTB/RIF—*Mycobacterium tuberculosis*/rifampicin; AUC—area under the curve; NA—not applicable.

The diagnostic utility of mNGS for TB was evaluated against the other methods available, including TB-DNA detection, Xpert MTB/RIF, AFS, IGRA, and mycobacterial culture. Concordance among all tests was low, with only seven patients testing positive across all platforms (Figure 3A). mNGS achieved the highest diagnostic sensitivity (86.00%), while maintaining strong specificity (93.30%) (Figure 3B). In contrast, the sensitivities of TB-DNA detection (70.21%, Figure 3C) and Xpert MTB/RIF (63.83%, Figure 3D) were lower. Although AFS (Figure 3E) and culture (Figure 3F) showed perfect specificity (100.00%), their clinical utility was limited by poor sensitivities (16.28% and 29.55%, respectively). IGRA exhibited moderate sensitivity (71.43%) and specificity (79.15%) (Figure 3G). Receiver Operating Characteristic (ROC) analysis confirmed that mNGS had optimal efficacy (area under the curve [AUC] = 0.877), followed by Xpert MTB/RIF (AUC = 0.870, *p* = 0.87) and TB-DNA detection (AUC = 0.848, *p* = 0.61), whereas AFS performed worst (AUC = 0.609, *p* < 0.0001) (Figure 4).

### 3.3. Co-Detected Pathogens Profiles

**Figure 5:**
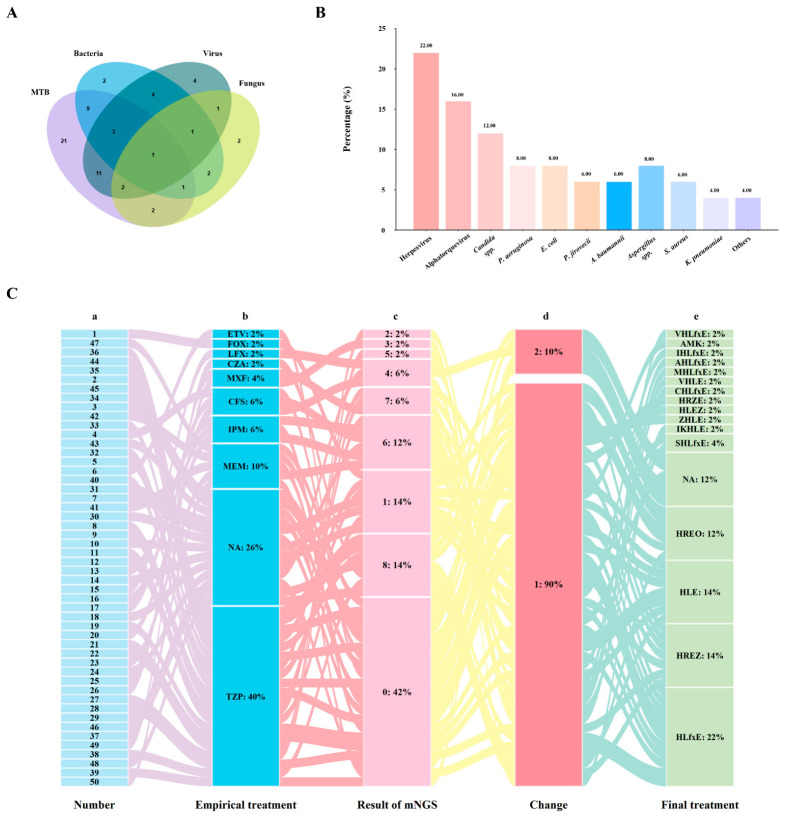
Pathogen profiles and changes in anti-infective treatment agents in AECOPD patients with TB. (**A**) Venn diagram of major pathogen categories detected by mNGS. (**B**) Constituent ratio of specific pathogen species detected by mNGS. (**C**) Flowchart of changes in anti-infective regimens before and after mNGS testing in patients with tuberculosis. Column a indicates the patient number. Column b represents empirical treatment, and Column c shows the mNGS results. The coding scheme for Column c is as follows: 0 = TB; 1 = TB + viruses; 2 = TB + fungi; 3 = TB + fungi+ bacteria; 4 = TB + fungi + viruses; 5 = TB + fungi+ bacteria+ viruses; 6 = TB+ bacteria+ viruses; 7= TB + *Pneumocystis jirovecii*; 8 = Not applicable. Column d indicates whether the initial treatment plan was modified (1 = Yes; 2 = No). Column e indicates the final treatment plan. Abbreviations: TB—tuberculosis; AECOPD—acute exacerbations of chronic obstructive pulmonary disease; mNGS—metagenomic next-generation sequencing; MTB—*Mycobacterium tuberculosis*; *P. aeruginosa*—*Pseudomonas aeruginosa*; *E. coli*—*Escherichia coli*; *P. jirovecii*—*Pneumocystis jirovecii*; *A. baumannii*—*Acinetobacter baumannii*; *S. aureus*—*Staphylococcus aureus*; *K. pneumoniae*—*Klebsiella pneumoniae*; NA—not applicable; ETV—entecavir; FOX—cefoxitin; LFX—levofloxacin; CZA—ceftazidime–avibactam; MXF—moxifloxacin; CFS—cefoperazone–sulbactam; IPM—imipenem; MEM—meropenem; TZP—piperacillin–tazobactam; AMK—amikacin. VHLfxE—voriconazole + isoniazid + rifapentine + levofloxacin + ethambutol; AHLfxE—amikacin + isoniazid + rifapentine + levofloxacin + ethambutol; MHLfxE—meropenem + isoniazid + rifapentine + levofloxacin + ethambutol; VHLE—voriconazole + isoniazid + rifapentine + ethambutol; CHLfxE—caspofungin + isoniazid + rifapentine + levofloxacin + ethambutol;; SHLfxE—sulfamethoxazole + isoniazid + rifapentine + levofloxacin + ethambutol; HREZ—isoniazid + rifampicin + ethambutol + pyrazinamide; HLE—isoniazid + rifapentine + ethambutol; HLfxE—isoniazid + rifapentine + levofloxacin + ethambutol; HREO—isoniazid + rifampicin+ ethambutol + ofloxacin.

Additionally, mNGS broadly identified a spectrum of co-detected pathogens (58.00%, 29/50), including viruses (54.00%, 27/50), bacteria (46.00%, 23/50), and fungi (24.00%, 12/50) (Figure 5A). Specifically, Herpesvirus was most common (22.00%), followed by Alphatorquevirus (16.00%) and *Candida* spp. (12.00%). Other frequently co-detected organisms included *Pseudomonas aeruginosa*, *Escherichia coli*, and *Aspergillus* spp. (each 8.00%), as well as *Acinetobacter baumannii*, *Staphylococcus aureus*, and *Pneumocystis jirovecii* (each 6.00%) (Figure 5B). Although no anti-tuberculosis therapy had been initiated prior to testing, it was subsequently started in 90.00% (45/50) of patients based on the mNGS report. Furthermore, of the other pathogens co-detected by mNGS, 31.03% (9/29) were treated with corresponding antimicrobials (Figure 5C). These nine co-infected cases were excluded from the intergroup analysis to avoid confounding effects.

### 3.4. Correlation Between mNGS Reads and Other Indicators

**Figure 6:**
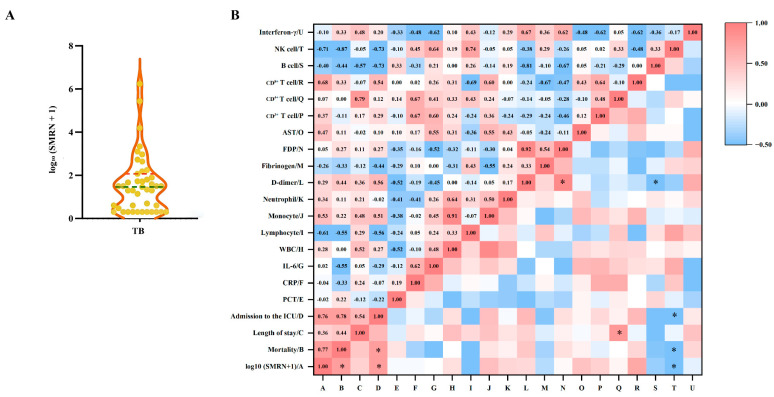
SMRN distribution and its correlation with clinical prognosis and laboratory indicators in AECOPD patients with TB. (**A**) Distribution of log_10_-transformed SMRN (log_10_(SMRN+1)). The dots represent the value of log_10_(SMRN+1) for each case. The green dashed line represents the median, while the red dashed lines represent the lower and upper quartiles (Q1 and Q3) of the log_10_(SMRN+1) values. (**B**) Correlation heatmap between log_10_(SMRN+1) and clinical prognosis as well as laboratory indicators. The figure in the box represents the Spearman correlation coefficient (r). Red boxes represent positive correlations, and blue boxes represent negative associations. Symbol * indicates a statistically significant correlation between two variables (*p* < 0.05). Abbreviations: SMRN—stringently mapped read number; ICU—intensive care unit; PCT—procalcitonin; CRP—C-reactive protein; IL-6—Interleukin-6; WBC—white blood cell count; FDP—fibrin(ogen) degradation products; AST—aspartate aminotransferase; T Cell—T lymphocyte; B cell—B lymphocyte; NK cell—natural killer cell.

Stringently mapped reads number (SMRN) distribution in the TB group was visualized using a log_10_ (SMRN + 1) transformation (Figure 6A). Most samples clustered at low read counts (log_10_ (SMRN + 1) < 2, corresponding to <99 raw reads), while a subset exhibited extremely high read counts (log_10_ (SMRN + 1) > 4, corresponding to >9999 raw reads). The median line was positioned at a low level (log_10_ (SMRN + 1) =1.8, corresponding to 63 raw reads). A correlation heatmap (Figure 6B) demonstrated that log_10_ (SMRN + 1) correlated strongly and positively with ICU admission rate (r = 0.76) and in-hospital mortality (r = 0.77). Conversely, strong negative correlations were observed with NK cell (r = −0.71) counts.

### 3.5. Risk Factors for TB Occurrence

**Figure 7:**
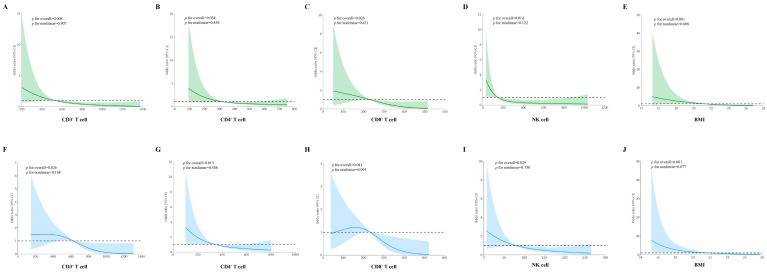
Restricted cubic spline analysis of immune cell counts and BMI in relation to TB vs. bacterial infection and TB vs. control group. (**A**) CD3^+^ T Cell count and risk of TB vs. bacterial *Infect*. (**B**) CD4^+^ T Cell count and risk of TB vs. bacterial infection; (**C**) CD8^+^ T Cell count and risk of TB vs. bacterial *Infect*. (**D**) NK Cell count and risk of TB vs. bacterial infection (**E**) BMI and risk of TB vs. bacterial *Infect*. (**F**) CD3^+^ T Cell count and risk of TB vs. control group. (**G**) CD4^+^ T Cell count and risk of TB vs. control group. (**H**) CD8^+^ T Cell count and risk of TB vs. control group. (**I**) NK Cell count and risk of TB vs. control group. (**J**) BMI and risk of TB vs. control group. The black dash lines indicates an odds ratio of 1. Abbreviations: BMI—body mass index; TB—tuberculosis; T Cell—T lymphocyte; NK cell—natural killer cell.

The restricted cubic spline analysis further revealed that CD3^+^, CD4^+^, CD8^+^ T cell counts, NK cells, and BMI displayed linear negative associations with the occurrence of TB compared to the bacterial group (Figure 7A–E) and control infection (Figure 7F–J) (all *p* for overall < 0.05, *p* for nonlinear > 0.05). The logistic regression analysis confirmed only reduced NK cell levels and lower BMI as independent risk factors for TB occurrence (Table 2).

## 4. Discussion

AECOPD triggered by TB is consistently associated with more severe clinical manifestations and a poorer prognosis. Our present study confirmed that the TB group experienced significantly longer hospital stays and had the highest ICU admission rates and the longest ICU duration. Therefore, achieving a timely and accurate etiological diagnosis remains a formidable challenge.

Conventional diagnostic approaches for TB (e.g., smearing, culture) are limited by long processing times and low positivity rates [30]. In contrast, nucleic acid-based molecular tests are extensively used for TB diagnosis owing to their superior specificity and sensitivity [31]. Furthermore, nucleic acid amplification tests (such as Xpert MTB/RIF Ultra) are the initial diagnostic test for patients with suspected TB [32]. For TB diagnosis, qPCR demonstrated a sensitivity of 0.66 (95% CI = 0.60–0.71) and a specificity of 0.98 (95% CI = 0.97–0.99) [33]. Xpert MTB/RIF assay showed a sensitivity of 87.2% (95% CI = 82.5–90.8%) and a specificity of 96.5% (95% CI = 95.1–97.5%) [34]. As a novel molecular diagnostic approach, mNGS demonstrated good diagnostic performance for pulmonary infections. In our study, mNGS exhibited high diagnostic efficacy for TB in AECOPD patients, achieving a sensitivity of 86.00% and a specificity of 93.30%. A recent meta-analysis reported that mNGS achieved a pooled sensitivity of 83% (95% CI = 69–91%) and specificity of 99% (95% CI = 92–100%) for pulmonary TB, compared with 72% (95% CI = 53–85%) and 100% (95% CI = 100–100%), respectively, for Xpert MTB/RIF [35]. In BALF samples from 236 patients with suspected pulmonary infections, mNGS yielded a sensitivity of 73.33% (95% CI = 58–89%) and specificity of 98.54% (95% CI = 97–100%) for TB detection [36]. In comparison, PCR demonstrated a sensitivity of 53.33% (95% CI = 35–71%) and a specificity of 100% (95% CI = 100–100%), while Xpert MTB/RIF showed a sensitivity of 60.00% (95% CI = 42–78%) and a specificity of 100% (95% CI = 100–100%) [36].

Notably, substantial heterogeneity in the diagnostic performance of mNGS for TB has been reported in previous studies. A study using BALF from patients with suspected TB and community-acquired pneumonia reported a sensitivity of only 47.92% [37]. In contrast, another study using formalin-fixed paraffin-embedded tissue from postoperative patients reported significantly higher performance, with 100.00% sensitivity and 88.24% specificity [38]. This marked variability can be attributed to several factors. Firstly, sample type intrinsically influences detection efficiency; lung tissue biopsies generally yield superior mNGS performance compared to BALF [39]. Secondly, the host’s immune status and the microbial load within the specimen significantly impact sequencing yields [40]. Finally, technical variations in sequencing platforms, depth, and bioinformatic thresholds also contribute to these discrepancies [41].

More importantly, the interpretation of mNGS results requires careful evaluation. Considering the potential background microbial DNA contamination during BALF sample processing, positive mNGS results should be defined under rigorous quality-control procedures. Also, positive mNGS results should be interpreted based on a comprehensive evaluation together with patients’ clinical manifestations, radiological findings, laboratory indicators, and other microbiological results to determine the causative pathogens. Furthermore, combining mNGS with conventional diagnostic methods may improve the overall performance and clinical applicability. Nevertheless, despite its promising diagnostic performance, the routine implementation of mNGS in public health systems and resource-limited settings remains challenging because of its relatively high cost, technical complexity, and dependence on specialized bioinformatic analysis [42]. Therefore, mNGS may currently be more suitable for patients with suspected complicated infections.

Furthermore, in our study, mNGS identified that 18.00% of AECOPD patients with TB had concurrent infections with other pathogens. Other studies indicated that polymicrobial infections occur in 15~40% of AECOPD patients and can exceed 50% in severe cases [43], with bacterial–viral co-infections being the most frequent pattern [44]. Such mixed infections were the drivers of increased disease severity and mortality [44]. Our findings highlight the significant clinical utility of mNGS as a comprehensive diagnostic tool, particularly for uncovering complex polymicrobial infections that may be missed by conventional methods in the management of AECOPD. Therefore, for AECOPD patients presenting with fever of unknown origin or diagnostically challenging conditions, mNGS may provide important clinical value through broad-spectrum pathogen screening within a relatively short turnaround time. Importantly, chronic obstructive pulmonary disease (COPD) and lung cancer were reported to frequently coexist [45]; accordingly, mNGS-based analysis of malignancy-associated indicators may provide additional value for clinical decision-making and potentially improve patient outcomes in COPD patients. Moreover, the capability of mNGS to sensitively detect the viral genomic sequences also enables its potential application in “One Health” surveillance through the early identification and monitoring of emerging or re-emerging viral pathogens [46].

In addition, we also assessed the clinical relevance of SMRN with other indicators in AECOPD patients with TB. We found that a higher SMRN was positively correlated with in-hospital mortality and ICU admission, but negatively correlated with host immune cell counts. A previous study suggested that mNGS read counts can correlate with the disease severity or prognosis in specific infections like endophthalmitis and sepsis [47]. These findings indicate that the SMRN could serve as a valuable biomarker to help stratify disease severity and estimate pathogen burden, particularly in immunocompromised patients.

Finally, we identified a reduced NK cell count and a lower BMI as independent risk factors of TB occurrence in AECOPD patients. These findings shift the focus from pharmacological agents to host-specific vulnerabilities, underscoring the critical role of the individual’s immune and nutritional status in predisposing to secondary TB. NK cells are pivotal effector cells in anti-TB immunity, protecting the host by directly lysing infected cells and secreting key cytokines [48]. However, in active TB, NK cell function is often impaired due to exhaustion [49]. Concurrently, low BMI is a well-documented risk factor for TB [50,51], intrinsically linked to systemic immune dysregulation. In individuals with latent TB, low BMI is associated with broad disruptions in immune cell subsets [52]. Transcriptomic analyses further reveal that under-nutrition drives a peripheral immune response characterized by hyper-activated inflammatory pathways and gene signatures associated with TB progression [53]. Thus, nutritional depletion and the consequent immune dysfunction appear to synergistically predispose AECOPD patients to TB. Therefore, TB-related diagnostic testing should be considered in AECOPD patients, particularly in those with low BMI and reduced NK cell counts.

This study has several limitations. As a retrospective single-center study, selection and information biases were unavoidable, and confounding factors could not be fully adjusted. The small sample size may reduce statistical power, and the lack of long-term follow-up precluded prognostic evaluation. Further large prospective studies are required to validate our findings.

## 5. Conclusions

In summary, mNGS is a robust diagnostic approach for TB diagnosis and concurrent mixed infections in AECOPD patients. Lower BMI and reduced NK cell counts were identified as independent risk factors for TB in this cohort. Therefore, integrating mNGS into the diagnostic workflow for AECOPD patients with immuno-nutritional impairment is recommended to enable timely, targeted treatment and improve clinical outcomes.

## Figures and Tables

**Table 1:** Baseline and clinical characteristics of AECOPD patients classified into tuberculosis, bacterial infection, and control groups.

Variables	TB (*n* = 41)	Bacterial Infection (*n* = 73)	Control (*n* = 56)	*p* Value ^#^
**Baseline characteristics**				
Age (years), median (Q_1_, Q_3_)	68.00 (59.00, 75.00)	72.00 (65.00, 77.00)	70.50 (60.75, 76.25)	0.17
Male, *n* (%)	37 (84.09)	55 (75.34)	42 (75.00)	0.17
BMI (kg/m^2^), median (Q_1_, Q_3_)	19.96 (17.19, 22.49)	23.15 (18.89, 25.32)	23.62 (21.30, 25.25)	<0.01 *
Smoke, *n* (%)	28 (68.29)	43 (59.72)	28 (50.00)	0.17
**Medication history, *n* (%)**				
LAMA/LABA	14 (34.14)	32 (43.84)	20 (35.71)	0.20
Budesonide-formoterol	7 (17.07)	13 (17.81)	16 (28.57)	0.14
Other hormones	5 (12.19)	7 (9.59)	10 (17.86)	0.36
**Laboratory** **findings, median (Q_1_, Q_3_)**				
PCT (ng/mL)	0.10 (0.05, 0.25)	0.15 (0.05, 0.62)	0.08 (0.04, 0.16)	0.02 *
CRP (mg/L)	50.55 (13.61, 91.10)	18.15 (5.42, 74.40)	12.15 (4.07, 36.38)	0.01 *
IL-6 (pg/mL)	26.95 (14.30, 59.47)	11.50 (6.07, 38.50)	19.20 (8.23, 26.88)	0.06
WBC (×10^9^/L)	6.46 (4.87, 8.47)	8.42 (6.51, 11.67)	6.30 (5.23, 7.64)	<0.01 *
Neutrophil (×10^9^/L)	4.72 (3.32, 6.44)	6.33 (4.12, 9.37)	4.59 (3.20, 5.78)	<0.01 *
Lymphocyte (×10^9^/L)	1.00 (0.42, 1.50)	1.06 (0.72, 1.59)	1.11(0.86, 1.58)	0.21
Monocyte (×10^9^/L)	0.55 (0.42, 0.67)	0.67 (0.51, 0.87)	0.50 (0.39, 0.62)	<0.01 *
CD3+ T cell count (/μL)	440.00 (306.00, 677.00)	714.50 (465.00, 931.00)	652.00 (515.00, 1092.00)	<0.01 *
CD4+ T cell count (/μL)	222.00 (134.00, 371.00)	387.00 (258.00, 538.25)	349.00 (309.00, 526.00)	<0.01 *
CD8+ T cell count (/μL)	201.00 (97.50, 243.00)	277.00 (168.00, 375.50)	255.00 (179.00, 447.00)	<0.01 *
CD4+/CD8+ T cell	1.50 (0.91, 2.72)	1.64 (1.04, 2.10)	1.43 (1.05, 1.84)	0.71
NK cell count (/μL)	105.00 (65.00, 147.00)	233.00 (135.00, 325.00)	253.00 (164.50, 340.00)	<0.01 *
B cell count (/μL)	60.00 (39.00, 104.00)	87.00 (45.00, 140.00)	92.50 (91.75, 93.25)	0.26
D-dimer (mg/L)	1.98 (1.01, 4.96)	1.44 (0.60, 3.12)	1.12 (0.55, 2.22)	0.01 *
FDP (mg/L)	6.40 (2.90, 12.60)	3.80 (2.50, 7.65)	2.90 (2.50, 5.50)	<0.01 *
AST (U/L)	25.00 (18.00, 33.75)	20.00 (16.00, 28.00)	17.00 (12.25, 25.00)	<0.01 *
Crea (U/L)	63.50 (57.00, 77.75)	83.00 (65.00, 114.25)	68.00 (58.25, 86.00)	<0.01 *
**Clinical outcome**				
In-hospital mortality, *n* (%)	6 (14.63)	10 (13.70)	5 (8.93)	0.65
Length of stay (days), median (Q_1_, Q_3_)	19.50 (14.25, 24.00)	15.00 (12.00, 22.00)	13.00 (11.00, 17.00)	<0.01 *
Length of admission to ICU (days), median (Q_1_, Q_3_)	0.00 (0.00, 10.75)	0.00 (0.00, 0.00)	0.00 (0.00, 0.00)	<0.01 *
Admission to the ICU, *n* (%)	19 (46.34)	11 (15.07)	2 (3.57)	<0.01 *

Abbreviations: AECOPD—acute exacerbations of chronic obstructive pulmonary disease; TB—tuberculosis; Q1—first quartile; Q3—third quartile; BMI—body mass index; LAMA/LABA—umeclidinium bromide/vilanterol trifenatate inhalation powder; PCT—procalcitonin; CRP—C-reactive protein; IL-6—interleukin-6; WBC—white blood cell count; T cell—T lymphocyte; NK cell—natural killer cell; B cell—B lymphocyte; FDP—fibrin(ogen) degradation products; AST—aspartate aminotransferase; Crea—creatinine; ICU—intensive care unit. ^#^ Statistical significance between the characteristics of the three groups was analyzed using the Chi–square test or Kruskal–Wallis test. *p*-values < 0.05 are considered statistically significant. The asterisk (*) indicates statistically significant *p*-values.

**Table 2:** Logistic regression analysis of risk factors for TB occurrence in AECOPD patients.

Variables	Univariate Logistic Regression Analysis				Multivariable Logistic Regression Analysis			
	TB vs. Bacterial Infection		TB vs. Control		TB vs. Bacterial Infection		TB vs. Control	
	*p* Value	OR (95% CI)	*p* Value	OR (95% CI)	*p* Value	OR (95% CI)	*p* Value	OR (95% CI)
CD3^+^ T cell	<0.01	0.99 (0.99~0.99)	<0.01 *	0.99 (0.99~0.99)	0.47	0.99 (0.98~1.01)	0.52	0.99 (0.98~1.01)
CD4^+^ T cell	<0.01	0.99 (0.99~0.99)	<0.01 *	0.99 (0.99~0.99)	0.66	1.00 (0.99~1.02)	0.72	1.00 (0.99~1.02)
CD8^+^ T cell	<0.01	0.99 (0.99~0.99)	<0.01 *	0.99 (0.99~0.99)	0.87	1.00 (0.98~1.02)	0.74	1.00 (0.98~1.02)
NK cell	0.02	0.99 (0.99~0.99)	0.03 *	0.99 (0.99~0.99)	<0.01 *	0.99 (0.99~0.99)	0.04 *	0.99 (0.99~0.99)
BMI	<0.001	0.73 (0.62~0.86)	0.01 *	0.82 (0.70~0.95)	<0.01 *	0.70 (0.57~0.86)	0.02 *	0.79 (0.61~1.01)

*p*-values < 0.05 are considered statistically significant. The asterisk (*) indicates statistically significant *p*-values. Abbreviations: TB—tuberculosis; AECOPD—acute exacerbations of chronic obstructive pulmonary disease; OR—odds ratio; CI—confidence interval; T cell—T lymphocyte; NK cell—natural killer cell; BMI—body mass index.

## Data Availability

The datasets used and/or analyzed during the current study are available from the corresponding author on reasonable request.

## Supplementary Materials

The following supporting information can be downloaded at https://www.mdpi.com/article/10.3390/jcm15124507/s1: Figure S1: Clinical characteristics of AECOPD patients among the tuberculosis, bacterial infection and control groups; Figure S2: Distribution of chest CT findings and lesion sites among groups, along with pathological and radiological features in TB patients; Table S1: Laboratory results of AECOPD patients with TB, bacterial infection, and control groups.

## Abbreviations

AECOPD	Acute Exacerbations of Chronic Obstructive Pulmonary Disease
AFS	Acid-fast staining
AUC	Area Under the Curve
BALF	Bronchoalveolar lavage fluid
BMI	Body mass index
CI	Confidence interval
COPD	Chronic obstructive pulmonary disease
CRP	C-reactive protein
FDP	Fibrin(ogen) Degradation Products
GOLD	Global Initiative for Chronic Obstructive Lung Disease
IGRA	Interferon-gamma release assays
IL-6	Interleukin-6
mNGS	Metagenomic next-generation sequencing
MTB	*Mycobacterium tuberculosis*
NK cell	Natural killer cell
FOR	Odds ratio
PCT	Procalcitonin
qPCR	Real-time quantitative polymerase chain reaction
RIF	Rifampicin
SMRN	Stringently mapped read number
AST	Aspartate aminotransferase
Crea	Creatinine
ICU	Intensive care unit
T Cell	T lymphocyte
TB	Tuberculosis
WBC	White blood cell

## References

[1] de Oca M.M., Perez-Padilla R., Celli B., Aaron S.D., Wehrmeister F.C., Amaral A.F.S., Mannino D., Zheng J., Salvi S., Obaseki D. (2025). The global burden of COPD: Epidemiology and effect of prevention strategies. Lancet Respir. Med..

[2] Jacobs D.M., Noyes K., Zhao J., Gibson W., Murphy T.F., Sethi S., Ochs-Balcom H.M. (2018). Early Hospital Readmissions after an Acute Exacerbation of Chronic Obstructive Pulmonary Disease in the Nationwide Readmissions Database. Ann. Am. Thorac. Soc..

[3] Cao Y., Xing Z., Long H., Huang Y., Zeng P., Janssens J.P., Guo Y. (2021). Predictors of mortality in COPD exacerbation cases presenting to the respiratory intensive care unit. Respir. Res..

[4] Aung H.W.W., Vermeersch K., McAuley H.J.C., Ramakrishnan S., Abdo M., Beersaerts A., Cass S.P., Smallcombe N., Gyselinck I., Pott H. (2026). Multidimensional prognostic risk stratification of COPD exacerbations: The baseline, acuity, and trigger (BAt) model. Lancet Respir. Med..

[5] Waeijen-Smit K., Crutsen M., Keene S., Miravitlles M., Crisafulli E., Torres A., Mueller C., Schuetz P., Ringbæk T.J., Fabbian F. (2024). Global mortality and readmission rates following COPD exacerbation-related hospitalisation: A meta-analysis of 65 945 individual patients. ERJ Open Res..

[6] Sivakumaran S., Alsallakh M.A., Lyons R.A., Quint J.K., Davies G.A. (2023). Estimating the contribution of respiratory pathogens to acute exacerbations of COPD using routine data. J. Infect..

[7] Moghoofei M., Azimzadeh Jamalkandi S., Moein M., Salimian J., Ahmadi A. (2020). Bacterial infections in acute exacerbation of chronic obstructive pulmonary disease: A systematic review and meta-analysis. Infection.

[8] Wei Y.Y., Ye J.J., Zhang D.W., Hu L., Wu H.M., Fei G.H. (2024). Melatonin Rescues Influenza A Virus-Induced Cellular Energy Exhaustion via OMA1-OPA1-S in Acute Exacerbation of COPD. J. Pineal Res..

[9] Papaetis G.S., Anastasakou E., Tselou T., Sotiriou A., Rarra V.C., Roussou P., Karakatsani A., Orphanidou D. (2010). Serological evidence of Mycoplasma pneumoniae infection in patients with acute exacerbation of COPD: Analysis of 100 hospitalizations. Adv. Med. Sci..

[10] World Health Organization (2025). Global Tuberculosis Report 2025.

[11] Wang Z., Zhao S., Zhou Y., He Y. (2025). Assessing the Causal Relationship between Chronic Obstructive Pulmonary Disease and Tuberculosis: A Mendelian Randomization Study. Int. J. Chronic Obstr. Pulm. Dis..

[12] Hamada Y., Fong C.J., Copas A., Hurst J.R., Rangaka M.X. (2022). Risk for development of active tuberculosis in patients with chronic airway disease-a systematic review of evidence. Trans. R. Soc. Trop. Med. Hyg..

[13] Inghammar M., Ekbom A., Engstrom G., Ljungberg B., Romanus V., Lofdahl C.G., Egesten A. (2010). COPD and the risk of tuberculosis--a population-based cohort study. PLoS ONE.

[14] Kontsevaya I., Cabibbe A.M., Cirillo D.M., DiNardo A.R., Frahm N., Gillespie S.H., Holtzman D., Meiwes L., Petruccioli E., Reimann M. (2024). Update on the diagnosis of tuberculosis. Clin. Microbiol. Infect..

[15] Zaporojan N., Negrean R.A., Hodisan R., Zaporojan C., Csep A., Zaha D.C. (2024). Evolution of Laboratory Diagnosis of Tuberculosis. Clin. Pract..

[16] Rabello E., de-Paris F. (2026). Tuberculosis Diagnostic Methods: Clinical Applicability, Implementation Challenges, and Integrated Testing Strategies. Pathogens.

[17] Chin K.L., Anibarro L., Sarmiento M.E., Acosta A. (2023). Challenges and the Way forward in Diagnosis and Treatment of Tuberculosis Infection. Trop. Med. Infect. Dis..

[18] Chen S., Ouyang T., Wang K., Hou X., Zhang R., Li M., Zhang H., He Q., Li X., Liu Z. (2025). Application of metagenomic next-generation sequencing in pathogen detection of lung infections. Front. Cell. Infect. Microbiol..

[19] Chen H., Yin Y., Gao H., Guo Y., Dong Z., Wang X., Zhang Y., Yang S., Peng Q., Liu Y. (2020). Clinical Utility of In-house Metagenomic Next-generation Sequencing for the Diagnosis of Lower Respiratory Tract Infections and Analysis of the Host Immune Response. Clin. Infect. Dis..

[20] Gao Q., Li L., Su T., Liu J., Chen L., Yi Y., Huan Y., He J., Song C. (2024). A single-center, retrospective study of hospitalized patients with lower respiratory tract infections: Clinical assessment of metagenomic next-generation sequencing and identification of risk factors in patients. Respir. Res..

[21] Knox-Brown B., Patel J., Potts J., Ahmed R., Aquart-Stewart A., Cherkaski H.H., Denguezli M., Elbiaze M., Elsony A., Franssen F.M.E. (2023). Small airways obstruction and its risk factors in the Burden of Obstructive Lung Disease (BOLD) study: A multinational cross-sectional study. Lancet Glob. Health.

[22] Salvi S.S., Barnes P.J. (2009). Chronic obstructive pulmonary disease in non-smokers. Lancet.

[23] Miravitlles M., Auladell-Rispau A., Monteagudo M., Vázquez-Niebla J.C., Mohammed J., Nuñez A., Urrútia G. (2021). Systematic review on long-term adverse effects of inhaled corticosteroids in the treatment of COPD. Eur. Respir. Rev..

[24] Dong Y.H., Chang C.H., Wu F.L., Shen L.J., Calverley P.M.A., Lofdahl C.G., Lai M.S., Mahler D.A. (2014). Use of inhaled corticosteroids in patients with COPD and the risk of TB and influenza: A systematic review and meta-analysis of randomized controlled trials. a systematic review and meta-analysis of randomized controlled trials. Chest.

[25] Huang T.M., Kuo K.C., Wang Y.H., Wang C.Y., Lai C.C., Wang H.C., Chen L., Yu C.J., Perng D.-W., On the behalf of Taiwan Clinical Trial Consortium for Respiratory Diseases (TCORE) (2020). Risk of active tuberculosis among COPD patients treated with fixed combinations of long-acting beta2 agonists and inhaled corticosteroids. BMC Infect. Dis..

[26] Lee C.H., Lee M.C., Shu C.C., Lim C.S., Wang J.Y., Lee L.N., Chao K.M. (2013). Risk factors for pulmonary tuberculosis in patients with chronic obstructive airway disease in Taiwan: A nationwide cohort study. BMC Infect. Dis..

[27] Halpin D.M.G., Masekela R., Vogelmeier C.F., Ozoh O.B., Cruz A.A., Reddel H.K., Yorgancioglu A., Agusti A. (2026). Addressing the global challenges of COPD and asthma: A shared vision from the Global Initiative for Chronic Obstructive Pulmonary Disease (GOLD) and the Global Initiative for Asthma (GINA). Eur. Respir. J..

[28] Jones B.E., Ramirez J.A., Oren E., Soni N.J., Sullivan L.R., Restrepo M.I., Musher D.M., Erstad B.L., Pickens C., Vaughn V.M. (2026). Diagnosis and Management of Community-acquired Pneumonia: An Official American Thoracic Society Clinical Practice Guideline. Am. J. Respir. Crit. Care Med..

[29] de Blic J., Midulla F., Barbato A., Clement A., Dab I., Eber E., Green C., Grigg J., Kotecha S., Kurland G. (2000). Bronchoalveolar lavage in children. ERS Task Force on bronchoalveolar lavage in children. European Respiratory Society. Eur. Respir. J..

[30] Matteo M.J., Latini M.C., Martinovic D.N., Bottiglieri M. (2025). Update of diagnostic methods in tuberculosis (TB). Rev. Argent. Microbiol..

[31] Trajman A., Campbell J.R., Kunor T., Ruslami R., Amanullah F., Behr M.A., Menzies D. (2025). Tuberculosis. Lancet.

[32] Saukkonen J.J., Duarte R., Munsiff S.S., Winston C.A., Mammen M.J., Abubakar I., Acuna-Villaorduna C., Barry P.M., Bastos M.L., Carr W. (2025). Updates on the Treatment of Drug-Susceptible and Drug-Resistant Tuberculosis: An Official ATS/CDC/ERS/IDSA Clinical Practice Guideline. Am. J. Respir. Crit. Care Med..

[33] Meregildo-Rodriguez E.D., Asmat-Rubio M.G., Vasquez-Tirado G.A. (2023). Droplet digital PCR vs. quantitative real time-PCR for diagnosis of pulmonary and extrapulmonary tuberculosis: Systematic review and meta-analysis. Front. Med..

[34] Zhang M., Xue M., He J.Q. (2020). Diagnostic accuracy of the new Xpert MTB/RIF Ultra for tuberculosis disease: A preliminary systematic review and meta-analysis. Int. J. Infect. Dis..

[35] You Y., Ni Y.M., Shi G. (2024). Diagnostic accuracy of metagenomic next-generation sequencing in pulmonary tuberculosis: A systematic review and meta-analysis. Syst. Rev..

[36] Sun H., Chen Q., Zhang D., Hu L., Li S., Lu M., Wang Y., Su H., Gao Y., Guo J. (2025). Integrative study of pulmonary microbiome and clinical diagnosis in pulmonary tuberculosis patients. Microbiol. Spectr..

[37] Shi C.L., Han P., Tang P.J., Chen M.M., Ye Z.J., Wu M.Y., Shen J., Wu H.Y., Tan Z.Q., Yu X. (2020). Clinical metagenomic sequencing for diagnosis of pulmonary tuberculosis. J. Infect..

[38] Sun W.W., Dong Z.W., Zhou Y.M., Jin F., Liu H.C., Fan L. (2023). Improving the identification and diagnostic efficiency of Metagenomic Next-Generation Sequencing for mycobacterial granuloma on postoperative formalin-fixed paraffin-embedded specimens. Microbes Infect..

[39] Zhu N., Zhou D., Li S. (2021). Diagnostic Accuracy of Metagenomic Next-Generation Sequencing in Sputum-Scarce or Smear-Negative Cases with Suspected Pulmonary Tuberculosis. BioMed Res. Int..

[40] Xu J., Huang Q., Yu J., Liu S., Yang Z., Wang F., Shi Y., Li E., Li Z., Xiao Y. (2022). Metagenomic Next-Generation Sequencing for the Diagnosis of Suspected Opportunistic Infections in People Living with HIV. Infect. Drug Resist..

[41] Li Y., Bian W., Wu S., Zhang J., Li D. (2023). Metagenomic next-generation sequencing for Mycobacterium tuberculosis complex detection: A meta-analysis. Front. Public Health.

[42] Zhao Y., Du L., Song J., Sun W., Chen Y., Yu X., Huang H., Huang G., Huang E., Wang N. (2025). Hierarchical integration of mNGS, PCR, and other conventional methods for precision TB diagnostics. Microbiol. Spectr..

[43] Jahan R., Mishra B., Behera B., Mohapatra P.R., Praharaj A.K. (2021). Study of respiratory viruses and their coinfection with bacterial and fungal pathogens in acute exacerbation of chronic obstructive pulmonary diseases. Lung India.

[44] Sethi S., Murphy T.F. (2008). Infection in the pathogenesis and course of chronic obstructive pulmonary disease. N. Engl. J. Med..

[45] Forder A., Zhuang R., Souza V.G.P., Brockley L.J., Pewarchuk M.E., Telkar N., Stewart G.L., Benard K., Marshall E.A., Reis P.P. (2023). Mechanisms Contributing to the Comorbidity of COPD and Lung Cancer. Int. J. Mol. Sci..

[46] Russell T., Formiconi E., Casey M., McElroy M., Mallon P.W.G., Gautier V.W. (2025). Viral Metagenomic Next-Generation Sequencing for One Health Discovery and Surveillance of (Re)Emerging Viruses: A Deep Review. Int. J. Mol. Sci..

[47] Zhu J., Xia H., Tang R., Ng T.K., Yao F., Liao X., Zhang Q., Ke X., Shi T., Chen H. (2022). Metagenomic Next-Generation Sequencing Detects Pathogens in Endophthalmitis Patients. Retina.

[48] Esin S., Batoni G. (2015). Natural killer cells: A coherent model for their functional role in Mycobacterium tuberculosis infection. J. Innate Immun..

[49] Wang J., Chai Q., Lei Z., Wang Y., He J., Ge P., Lu Z., Qiang L., Zhao D., Yu S. (2024). LILRB1-HLA-G axis defines a checkpoint driving natural killer cell exhaustion in tuberculosis. EMBO Mol. Med..

[50] Arriaga M.B., Amorim G., Figueiredo M.C., Staats C., Kritski A.L., Cordeiro-Santo M., Rolla V.C., Rebeiro P.F., Andrade B.B., Sterling T.R. (2026). Body Mass Index and Incident Tuberculosis in Close Tuberculosis Contacts. Clin. Infect. Dis..

[51] Choi H., Yoo J.E., Han K., Choi W., Rhee S.Y., Lee H., Shin D.W. (2021). Body Mass Index, Diabetes, and Risk of Tuberculosis: A Retrospective Cohort Study. Front. Nutr..

[52] Rajamanickam A., Munisankar S., Dolla C.K., Babu S. (2019). Undernutrition is associated with perturbations in T cell-, B cell-, monocyte- and dendritic cell- subsets in latent Mycobacterium tuberculosis infection. PLoS ONE.

[53] VanValkenburg A., Kaipilyawar V., Sarkar S., Lakshminarayanan S., Cintron C., Prakash Babu S., Knudsen S., Joseph N.M., Horsburgh C.R., Sinha P. (2022). Malnutrition leads to increased inflammation and expression of tuberculosis risk signatures in recently exposed household contacts of pulmonary tuberculosis. Front. Immunol..

