# Supplementary material for: Metagenomic Next-Generation Sequencing for Pulmonary Tuberculosis Diagnosis and Infection Risk Factor Analysis in AECOPD Patients: A Single-Center Retrospective Study
Source: J Clin Med. 2026 Jun 10;15(12):4507. doi: 10.3390/jcm15124507 (PMC13301940; doi:10.3390/jcm15124507)
Supplement: Supplementary file 1 [file jcm-15-04507-s001.zip › jcm-4329613-supplementary.pdf]

# Supplemental materials

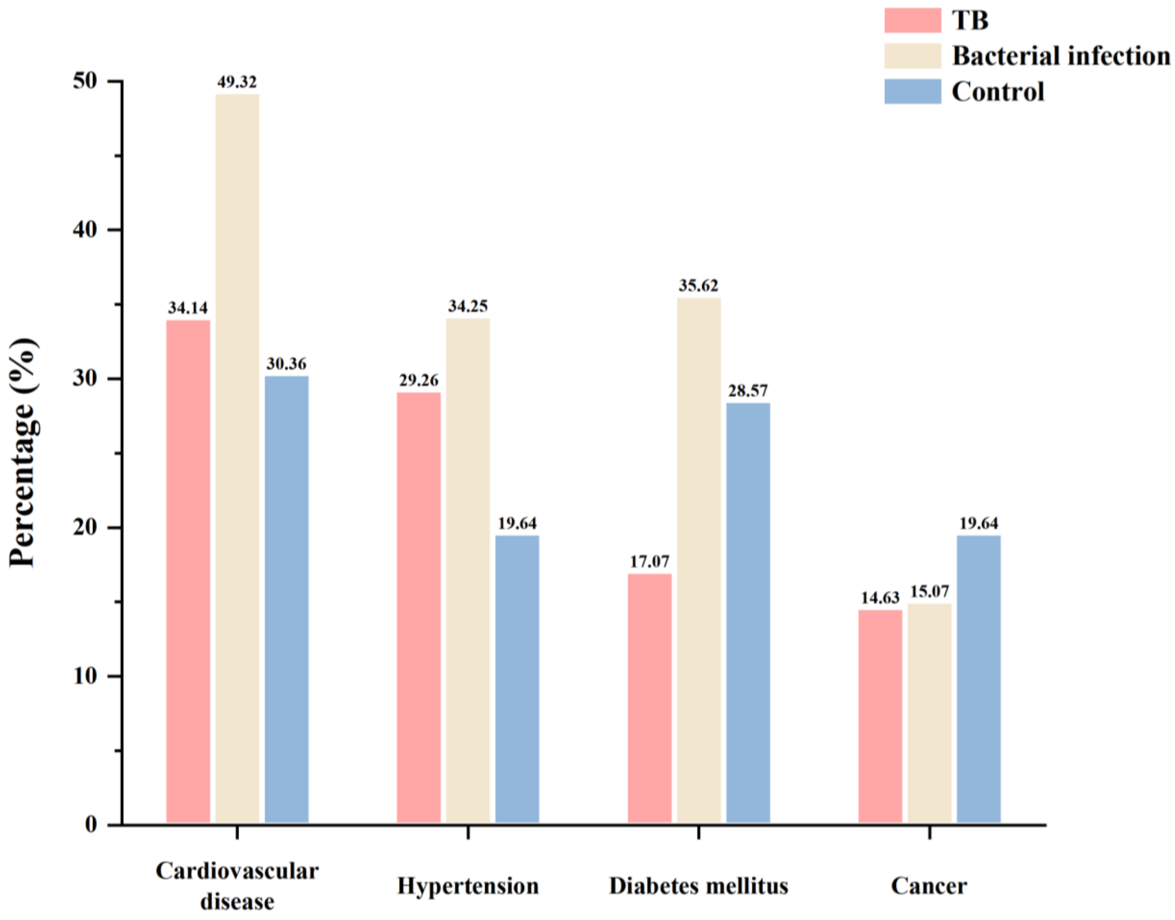


**Supplemental Figure S1.** Clinical characteristics of AECOPD patients among the tuberculosis, bacterial infection and control groups. Abbreviations: TB, tuberculosis.

A

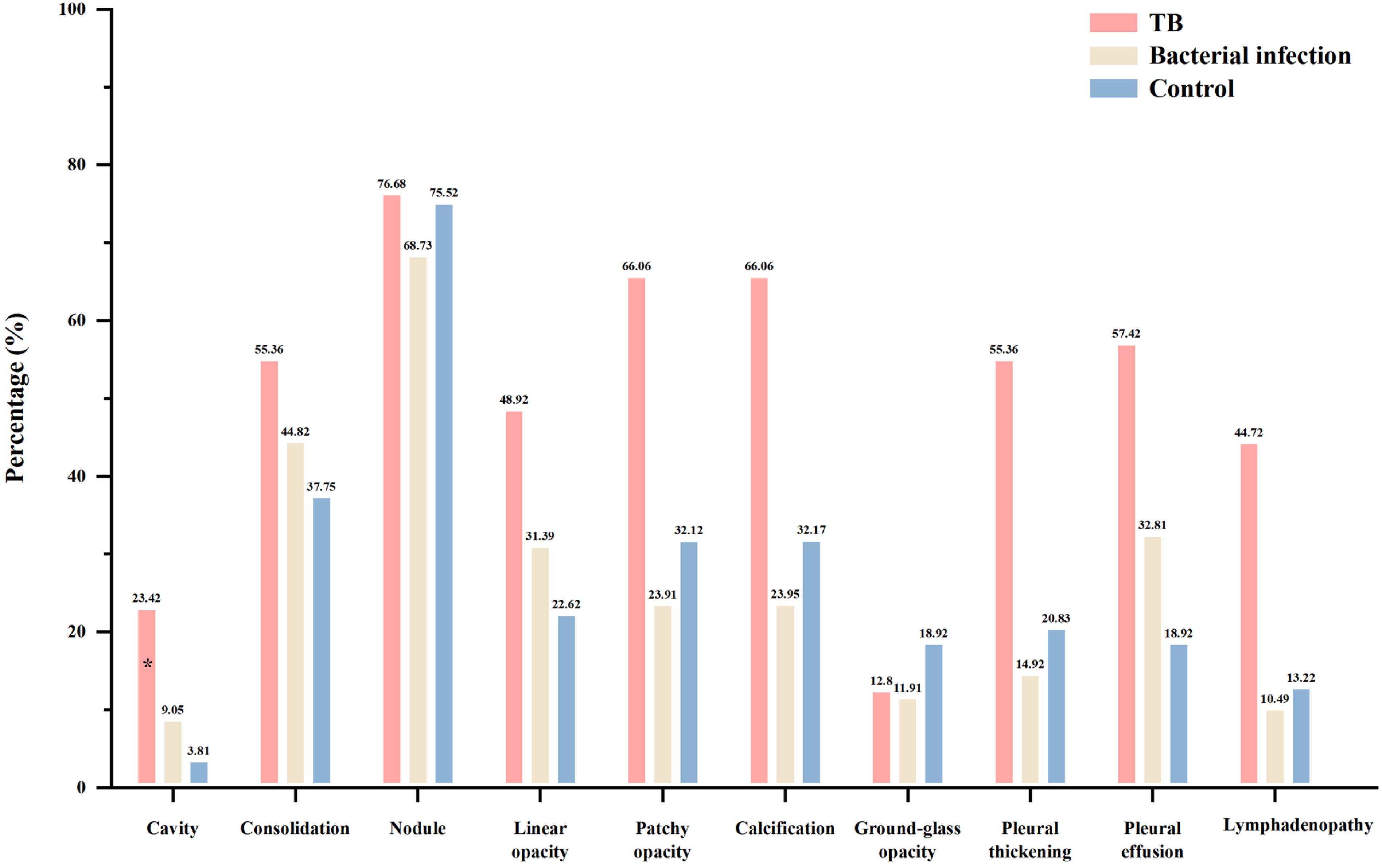
TB
Bacterial infection
Control
Percentage (%)
100
80
60
40
20
0
23.42
*
9.05
3.81
55.36
44.82
37.75
76.68
68.73
75.52
48.92
31.39
22.62
66.06
23.91
32.12
66.06
23.95
32.17
12.8
11.91
18.92
55.36
14.92
20.83
57.42
32.81
18.92
44.72
10.49
13.22
Cavity
Consolidation
Nodule
Linear opacity
Patchy opacity
Calcification
Ground-glass opacity
Pleural thickening
Pleural effusion
Lymphadenopathy

B

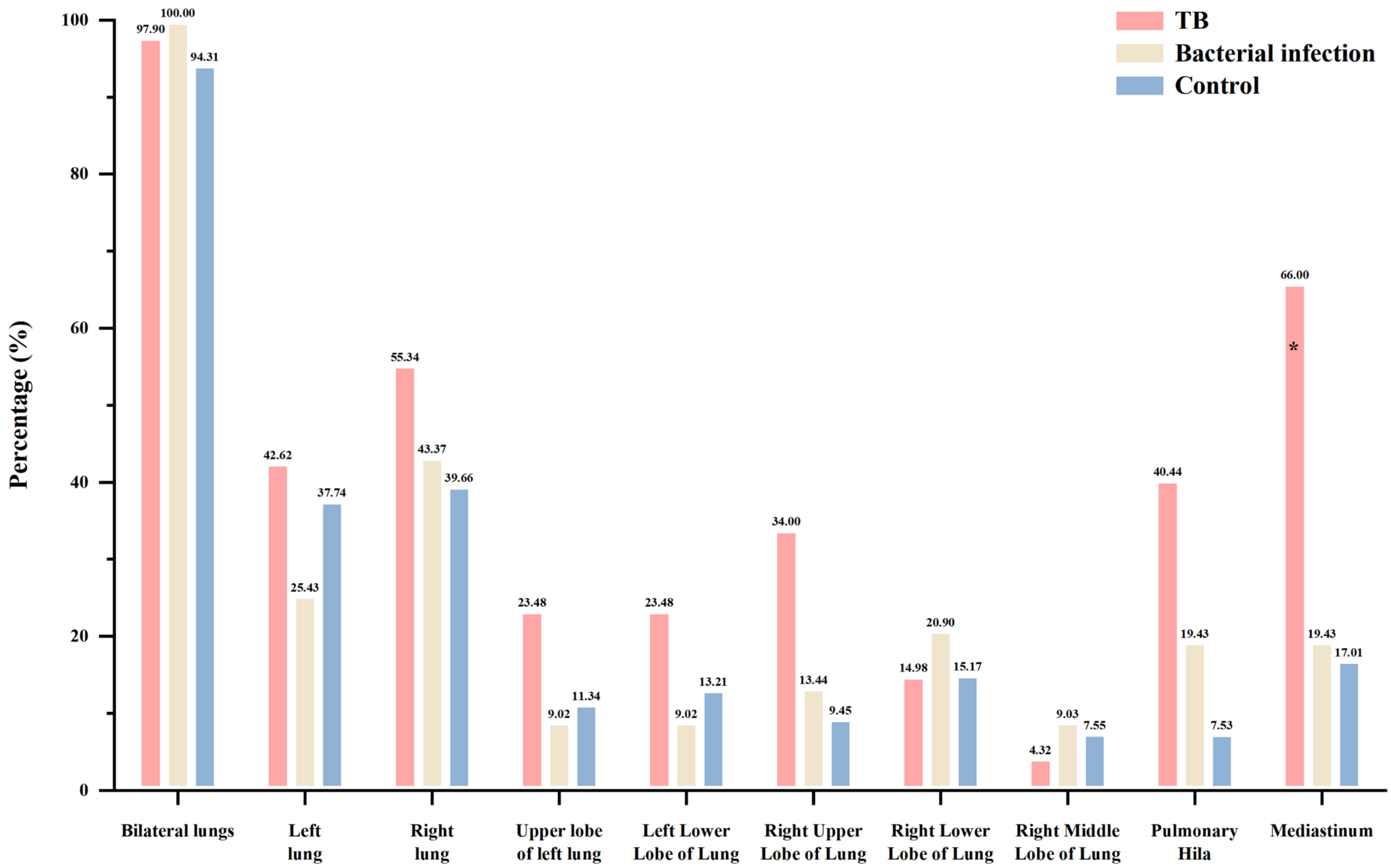
TB
Bacterial infection
Control
Percentage (%)
100
80
60
40
20
0
97.90
100.00
94.31
42.62
25.43
37.74
55.34
43.37
39.66
23.48
9.02
11.34
23.48
9.02
13.21
34.00
13.44
9.45
14.98
20.90
15.17
4.32
9.03
7.55
40.44
19.43
7.53
66.00
*
19.43
17.01
Bilateral lungs
Left lung
Right lung
Upper lobe of left lung
Left Lower Lobe of Lung
Right Upper Lobe of Lung
Right Lower Lobe of Lung
Right Middle Lobe of Lung
Pulmonary Hila
Mediastinum

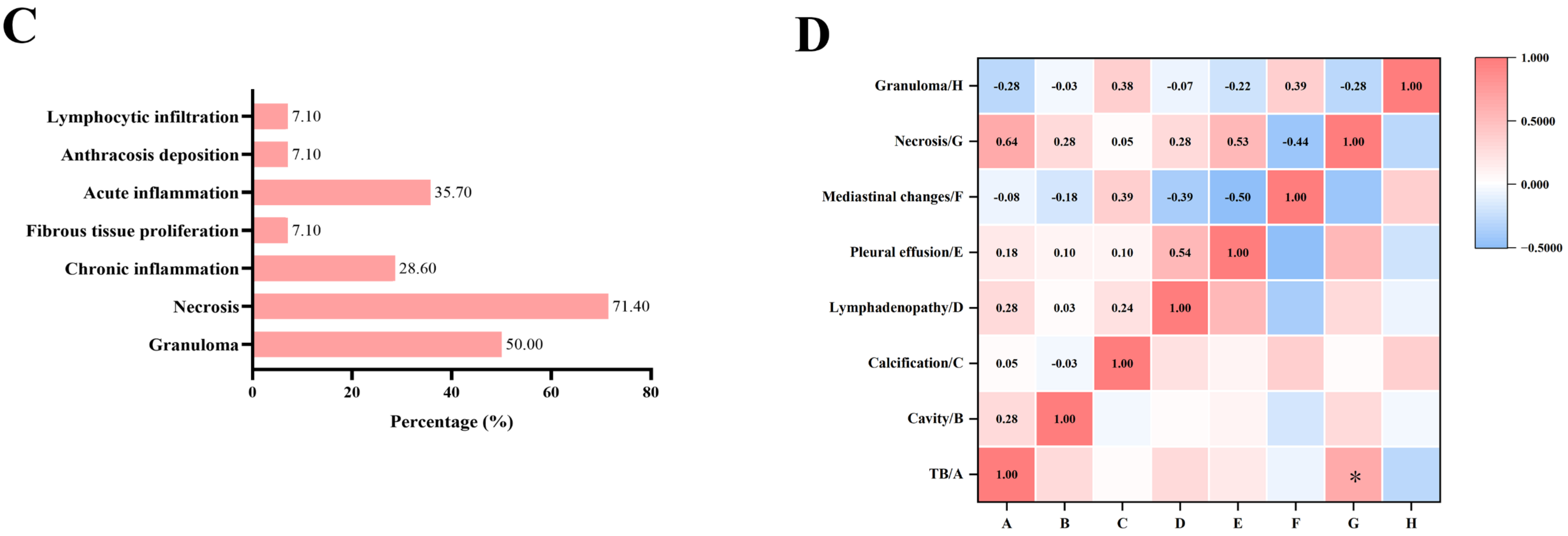


**Supplemental Figure S2.** Distribution of chest CT findings and lesion sites among groups, along with pathological and radiological features in TB patients. (A-B) Distribution of chest CT findings and lesion sites among TB, bacterial infection, and control groups. Symbol * indicates a statistically significant difference among the three groups (*P*<0.05). (C) Distribution of pathological characteristics in TB patients. (D) Correlation heatmap of radiological and pathological features in the patients with TB. The figure in the box represents the Spearman correlation coefficient (r). Red boxes represent positive correlations, and blue boxes represent negative associations. Symbol * indicates a statistically significant correlation between two variables (*P*<0.05). Abbreviations: CT, computed tomography; TB, tuberculosis.

**Supplement Table S1.** Laboratory results of AECOPD patients with TB, bacterial infection and control groups.

| **Indicators** | **TB (*n* = 50)** | **Control (*n* = 56)** | **Bacterial infection (*n* = 73)** | ***p* value** |
|---|---|---|---|---|
| Fibrinogen (mg/L), median ($Q_1$, $Q_3$) | 4.30 (3.36, 5.10) | 3.58 (2.82, 4.33) | 3.69 (3.03, 4.80) | 0.12 |
| Albumin (g/L), median ($Q_1$, $Q_3$) | 34.45 (31.55, 38.25) | 35.70 (31.90, 40.10) | 36.90 (33.18, 39.38) | 0.10 |
| ALT (U/L), median ($Q_1$, $Q_3$) | 19.50 (11.50, 24.75) | 19.50 (11.75, 27.25) | 19.00 (16.00, 25.00) | 0.14 |
| LDH (U/L), median ($Q_1$, $Q_3$) | 210.00 (167.00, 267.00) | 212.00 (180.00, 296.50) | 189.50 (158.00, 227.00) | 0.06 |
| $CD4^+/CD8^+$ T cell, median ($Q_1$, $Q_3$) | 1.50 (0.91, 2.72) | 1.64 (1.04, 2.10) | 1.43 (1.05, 1.84) | 0.71 |
| B cell count (/μL), median ($Q_1$, $Q_3$) | 60.00 (39.00, 104.00) | 87.00 (45.00, 140.00) | 92.50 (91.75, 93.25) | 0.26 |

*p*-values < 0.05 are considered statistically significant.

Abbreviations: Q1, first quartile; Q3, third quartile; TB, tuberculosis; ALT, alanine aminotransferase; LDH, lactate dehydrogenase; T cell, T lymphocyte; B cell, B lymphocyte.
